# Variability in Test Interval Is Linked to Glycated Haemoglobin (HbA1c) Trajectory over Time

**DOI:** 10.1155/2022/7093707

**Published:** 2022-05-16

**Authors:** Anthony A. Fryer, David Holland, Michael Stedman, Christopher J. Duff, Lewis Green, Jonathan Scargill, Fahmy W. F. Hanna, Pensée Wu, R. John Pemberton, Christine Bloor, Adrian H. Heald

**Affiliations:** ^1^School of Medicine, Keele University, Keele, Staffordshire, UK; ^2^Department of Clinical Biochemistry, University Hospitals of North Midlands NHS Trust, Stoke-on-Trent, Staffordshire, UK; ^3^The Benchmarking Partnership, Alsager, Cheshire, UK; ^4^Res Consortium, Andover, UK; ^5^Department of Clinical Biochemistry, St. Helens & Knowsley Teaching Hospitals NHS Trust, Whiston Hospital, Prescot, UK; ^6^Department of Clinical Biochemistry, The Royal Oldham Hospital, The Northern Care Alliance NHS Group, Oldham, UK; ^7^Department of Diabetes and Endocrinology, University Hospitals of North Midlands NHS Trust, Stoke-on-Trent, Staffordshire, UK; ^8^Centre for Health & Development, Staffordshire University, Staffordshire, UK; ^9^Department of Obstetrics & Gynaecology, University Hospitals of North Midlands NHS Trust, Stoke-on-Trent, Staffordshire, UK; ^10^Diabetes UK (North Staffordshire Branch), Porthill, Stoke-on-Trent, Staffordshire, UK; ^11^Department of Diabetes and Endocrinology, Salford Royal NHS Foundation Trust, Salford, UK; ^12^The School of Medicine and Manchester Academic Health Sciences Centre, The University of Manchester, Manchester, UK

## Abstract

**Aims:**

We previously showed that the glycated haemoglobin (HbA1c) testing frequency links to diabetes control. Here, we examine the effect of variability in test interval, adjusted for the frequency, on change in HbA1c (*Δ*HbA1c). *Materials & Methods*. HbA1c results were collected on 83,872 people with HbA1c results at baseline and 5 years (±3 months) later and ≥6 tests during this period. We calculated the standard deviation (SD) of test interval for each individual and examined the link between deciles of SD of the test interval and *Δ*HbA1c level, stratified by baseline HbA1c.

**Results:**

In general, less variability in testing frequency (more consistent monitoring) was associated with better diabetes control. This was most evident with moderately raised baseline HbA1c levels (7.0–9.0% (54–75 mmol/mol)). For example, in those with a starting HbA1c of 7.0–7.5% (54–58 mmol/mol), the lowest SD decile was associated with little change in HbA1c over 5 years, while for those with the highest decile, HbA1c rose by 0.4–0.6% (4–6 mmol/mol; *p* < 0.0001). Multivariate analysis showed that the association was independent of the age/sex/hospital site. Subanalysis suggested that the effect was most pronounced in those aged <65 years with baseline HbA1c of 7.0–7.5% (54–58 mmol/mol). We observed a 6.7-fold variation in the proportion of people in the top-three SD deciles across general practices.

**Conclusions:**

These findings indicate that the consistency of testing interval, not the just number of tests/year, is important in maintaining diabetes control, especially in those with moderately raised HbA1c levels. Systems to improve regularity of HbA1c testing are therefore needed, especially given the impact of COVID-19 on diabetes monitoring.

## 1. Introduction

The achievement and maintenance of adequate glycaemic control, as measured by glycosylated haemoglobin (HbA1c), are a focus of management strategies for patients with all forms of diabetes mellitus (DM) and involve the allocation of very significant resources worldwide. We previously described considerable variation in the proportion of people with DM achieving target control as measured by HbA1c in general practices in England [1, 2] in both type 1 diabetes (T1DM) and type 2 diabetes (T2DM).

Guidance from many professional/academic bodies worldwide has advocated regular HbA1c monitoring to optimise the chances of attaining treatment goals for people with diabetes. The American Diabetes Association guidelines [3] recommend testing “at least two times a year in patients who are meeting treatment goals (and who have stable glycaemic control)” and “quarterly in patients whose therapy has changed or who are not meeting glycaemic goals” whereas the UK National Institute for Health and Care Excellence guidance recommends measuring HbA_1c_ at “3–6-monthly intervals…, until the HbA_1c_ is stable on unchanging therapy” and “6-monthly intervals once the HbA_1c_ level and blood glucose lowering therapy are stable” [4, 5].

We and others have shown that many people with diabetes do not have tests at the recommended frequency [6–13]. Furthermore, poor adherence to guidelines on monitoring the frequency is not limited to diabetes. For example, studies have previously demonstrated significant variation in testing patterns in monitoring of thyroid stimulating hormone levels in people with hypothyroidism on thyroid hormone replacement therapy [14], in the management of gout [15] and in immunoglobulin testing by general practitioners [16]. Hence, testing frequency is recognised as an important factor in management of several long-term conditions.

The lack of concordance with the guidance on monitoring the frequency clearly has clinical implications. We previously described the way that the frequency of HbA1c testing relates to the outcome in terms of future HbA1c [17] and probability of achieving HbA1c targets [18], with the interval between HbA1c tests being an independent determinant of HbA1c control in people with diabetes. Others have also demonstrated the relation between numbers of tests per year and markers of glycaemic control, even after adjusting for factors such as age, gender, education level, and lifestyle markers [19, 20].

Although there is a significant body of evidence around the frequency of testing, there is a limited evidence base around how the pattern of HbA1c testing influences glycaemic control. We have used laboratory data to address the question: how does the pattern of HbA1c testing over time relate to changes in the HbA1c level, specifically by looking at the effect of *variability in test interval* on change in HbA1c over time? For example, while guidance recommended 4 tests per year, we hypothesised that the distribution of these tests across the year is also important.

## 2. Materials and Methods

We collected all HbA1c data from Laboratory Information and Management Systems from the University Hospitals of North Midlands (UHNM) (covering the Royal Stoke University and County Hospitals) and Pennine Acute Hospitals (PAT) (covering North Manchester General, Oldham, Rochdale and Bury Hospitals) NHS Trusts for the period 1 January 2012 to 31 December 2019. This comprised a total of 3,319,761 tests in 903,667 patients.

This study is part of an audit and quality improvement programme to increase the quality of laboratory test requesting. Hence, it includes a service evaluation and audit of local practice against the guidelines outlined by NICE [4, 5] with a view to increasing implementing quality improvements to enhance the clinical laboratory service. Accordingly, this study was not considered to be researched using the decision tool provided by the UK Health Research Authority [21] and did not require NHS Research Ethics Committee review. All data extracted from Laboratory Information and Management Systems were anonymised.

### 2.1. Selection of the Patient Cohort

The process for the selection of the study cohort is shown in Supplemental Figure S1. To standardise the effect of time on the change in HbA1c, we focused on people with a HbA1c test result within the first half of the study period who also had a HbA1c test 5 years (±3 months) later. This identified 2,173,215 tests in 341,165 patients. In cases where more than one pair of tests within a given patient met the criteria, the pair where the interval was closest to the 5 years was selected, leaving 946,452 tests in 126,509 patients.

As the study focused on variability in testing interval, we limited the study cohort to those patients who had at least 6 HbA1c tests during the 5-year period (including the baseline and final HbA1c test). This allowed us to assess the impact of variability on the testing interval on the change in HbA1c over a fixed time period.

The final study cohort comprised 802,153 tests in 83,872 people. The characteristics of these patients, split by the hospital site, are shown in Table 1.

### 2.2. Assessment of Variability in Testing Interval

In order to assess the impact of variability on the testing interval on the change in HbA1c over the 5-year period, we first calculated the standard deviation (SD) of the testing interval. To eliminate the effect of the number of tests between the start and end of the 5 years on the SD, we then calculated the deciles of the SD values separately for each group of patients with a given number of tests (i.e., separately for those with 6 tests, 7 tests, 8 tests, etc.). This was performed separately for the two hospital sites. It was not possible to calculate deciles with confidence for those with >20 tests (*n* = 660 patients) over the period due to the small numbers of patients in these groups (e.g., there were 209 patients with 21 tests, leaving only ~21 in each decile group). The deciles were then combined to give an overall assessment of variability in the testing interval, by the hospital site.

### 2.3. Assessment of Variation between General Practices

The baseline general practice code was available in 79,541 (94.8%) of patients. In those practices with at least 100 cases (76,822 patients), we calculated the proportion of cases within the three deciles with the highest variability in the HbA1c testing interval (SD deciles 8–10) for each practice. This was then represented as a ski-slope plot.

### 2.4. Data Analysis

For all statistical analyses, patients were stratified by baseline HbA1c: <4.9% (<30 mmol/mol), 5.0–5.9% (30–41 mmol/mol), 6.0–6.5% (42–47 mmol/mol), 6.5–7.0% (48–53 mmol/mol), 7.0–7.5% (54–58 mmol/mol), 7.5–9.0% (59–75 mmol/mol), 9.1–10.0% (76–86 mmol/mol), and >10.0% (>86 mmol/mol). These categories were based on commonly used decision points in the management of people with diabetes. Data from the two hospital sites were analysed separately to provide some independent assessment of the association between variability in testing interval and change in HbA1c over the 5-year period.

All statistical analysis was performed using Stata (version 14, College Station, TX). Associations between the change in HbA1c and the decile of SD in the testing interval, stratified by the category of baseline HbA1c, were assessed using linear regression, adjusted for a number of tests alone (testing frequency; as described above) and after further adjustment for other factors such as age and gender by inclusion in the regression model. A *p* value of <0.05 was considered statistically significant. The strength of the association between variability in the test interval and change on HbA1c over the 5-year period was determined using standard beta values.

## 3. Results

### 3.1. Patient Demographics

A description of the two cohorts (UHNM & PAT) is shown in Table 1. The UHNM patients were slightly older with a slightly higher median HbA1c and proportion of males. As expected, the median duration of follow-up was close to the expected 5 years for both sites and the median number of HbA1c tests over the follow-up period was the same. The distribution of patients by baseline HbA1c was different between the two sites with PAT having a higher proportion with lower baseline HbA1c values, particularly in the 5.0–5.9% (30–41 mmol/mol) category (Table 1).

### 3.2. Effect of Variability in the Testing Interval on the Change in HbA1c

**Figure 1:**
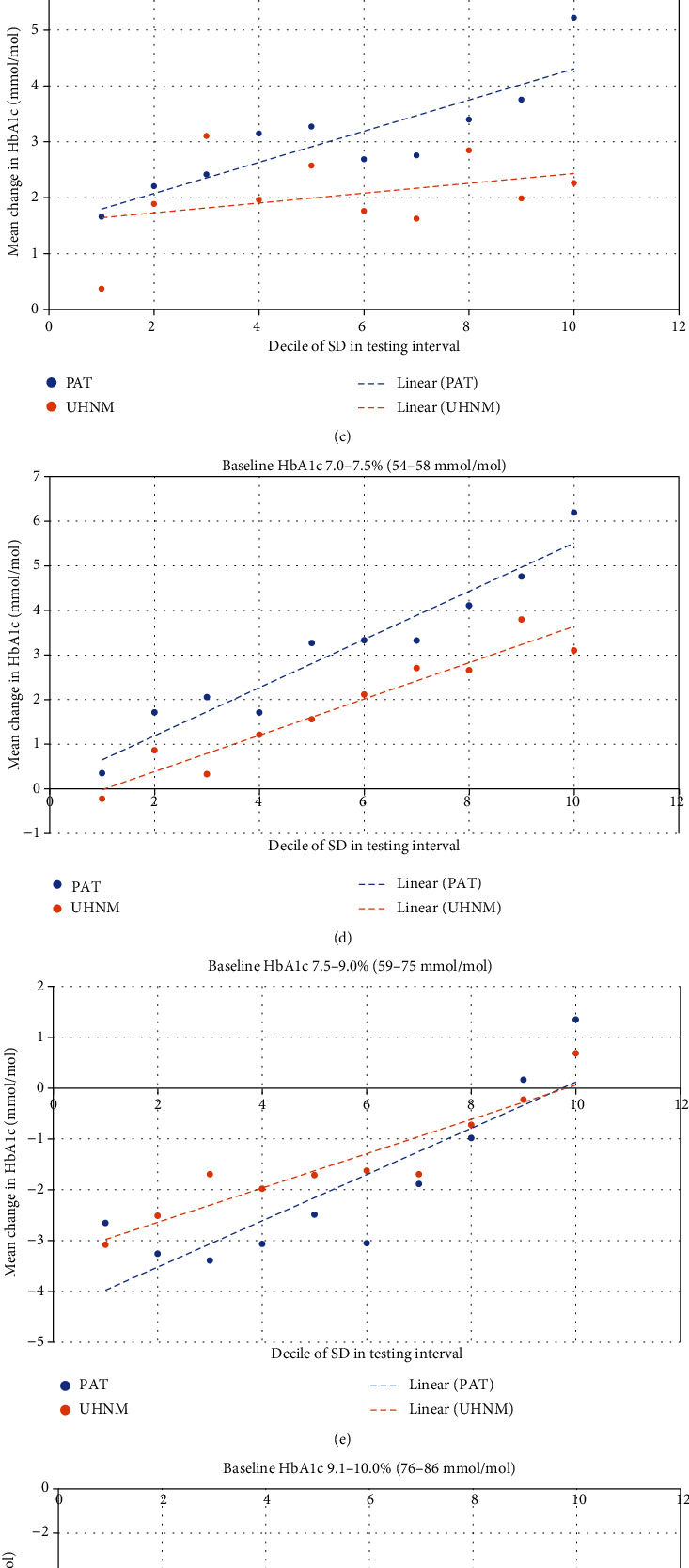
Association between availability in testing interval (expressed as decile of the test interval standard deviation) and change in HbA1c concentration between *t*_0_ and *t*_0+5 yrs_, stratified by baseline HbA1c: (a) 5.0–5.9% (30–41 mmol/mol), (b) 6.0–6.5% (42–47 mmol/mol), (c) 6.5–7.0% (48–53 mmol/mol), (d) 7.0–7.5% (54–58 mmol/mol), (e) 7.5–9.0% (59–75 mmol/mol), (f) 9.1–10.0% (76–86 mmol/mol), and (g) >10.0% (>86 mmol/mol).

We examined the variability, assessed as the decile of standard deviation (see Materials and Methods) across the two hospital sites, stratified by the baseline HbA1c category. When expressed as the mean change in HbA1c, we observed that there was an association between variability in the testing interval and change in HbA1c over the 5-year period across a number of baseline HbA1c categories for both sites (Figure 1).

For those with a baseline HbA1c of 5.0–5.9% (30–41 mmol/mol), those with SDs in the lowest decile demonstrated little change in HbA1c over the 5-year period, while those in the highest decile showed around a +0.4% (+4 mmol/mol) change in HbA1c, on average (Figure 1(a)). Deciles 2–9 showed intermediate increases in HbA1c in a broadly linear fashion. Linear regression showed that this was highly statistically significant for both sites (both <0.001; Table 2). A similar pattern was observed for those with a baseline HbA1c of 6.0–6.5% (42–47 mmol/mol), ranging from +0.1/+0.2% (+1/+2 mmol/mol) HbA1c change for the lowest SD decile (0.78 mmol/mol for UHNM, 1.66 mmol/mol for PAT) to +0.3/+0.4% (+3/+4 mmol/mol) for the highest decile (2.71 mmol/mol for UHNM, 4.33 mmol/mol for PAT), though the *Δ*HbA1c values were generally higher for PAT than UHNM (Figure 1(b)). The association was highly statistically significant at both hospital sites (both *p* < 0.001; Table 2), though the strength of the correlation, as reflected by the standard beta value, was lower than for the 5.0–5.9% (30–41 mmol/mol) group.

For the 6.5–7.0% (48–53 mmol/mol) group (Figure 1(c)), whilst the associations were generally similar, they were less strong for the UHNM cohort (*p* = 0.019) than PAT (*p* < 0.001).

For the 7.0–7.5% (54–58 mmol/mol) and 7.5–9.0% (59–75 mmol/mol) groups, the association between variability in testing interval and *Δ*HbA1c was particularly significant (all *p* < 0.0001; Figures 1(d) and 1(e)). Furthermore, the standard beta values suggest that variability in the testing interval accounted for ~10% of the variation in *Δ*HbA1c over the 5-year period. For the 7.5–9.0% (59–75 mmol/mol) group, the lowest decile was associated with a mean reduction on HbA1c of ~0.3% (~3 mmol/mol) while those cases with the highest decile saw a net increase in HbA1c of ~0.1% (~1 mmol/mol).

In the 9.1–10.0% (76–86 mmol/mol) baseline HbA1c category (Figure 1(f)), there was a similar significant association within the PAT cohort (*p* < 0.001) to that seen within other baseline HbA1c categories but the UHNM data just failed to achieve statistical significance (*p* = 0.068). There was no significant association observed in the >10.0% (>86 mmol/mol) group between variability of testing interval and *Δ*HbA1c (Figure 1(f)).

### 3.3. Effect of Demographic Factors

Using multivariate linear regression in the total case group, the association between variability in the testing interval and *Δ*HbA1c remained statistically significant after adjusting for age at baseline, sex, baseline HbA1c, and site (*p* < 0.001, standard beta = 0.03).

Given the similarities in results between the two sites, the data were combined and then used to further explore the effect of age and gender. Table 2 also shows the association between variability in testing interval and change in HbA1c stratified by age at baseline and sex. Associations in males and females appeared similar. However, with respect to age, those in the <65-year age group tended to have a stronger association than those in the older group (as expressed by standard beta values). This was particularly noticeable in those with higher baseline HbA1c values (≥7.0% [≥54 mmol/mol]), where the standard beta values in those aged <65 years were typically around 0.04 higher than in those aged 65 and over (Table 2). Furthermore, we also noted that those aged <65 years had a larger proportion of cases in deciles 8–10 (32.7%) than those aged ≥65 years (27.4%; *p* < 0.001, *χ*^2^_1_ = 192.8).

### 3.4. Variation in the Testing Pattern across General Practices

**Figure 2:**
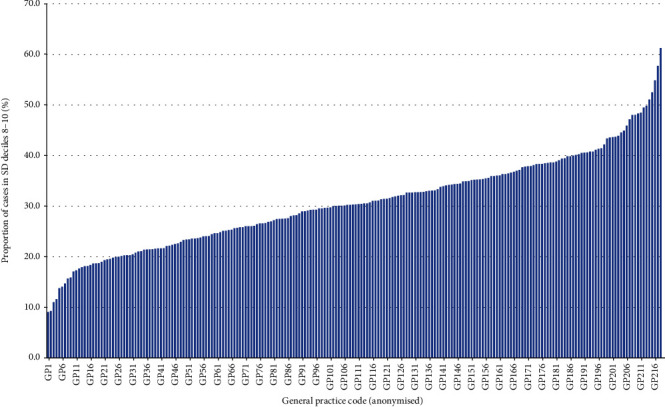
Variation in the proportion of individuals within the top-three deciles of standard deviation in HbA1c test intervals (highest variability in the test pattern) between 216 general practices.

We then examined the pattern of testing between individual general practices. We excluded those practices that had less than 100 cases and then, for each practice, calculated the proportion of cases within the top-three SD deciles (those with the most variability in testing interval). This showed that the proportion of cases within deciles 8–10 ranged from less than 10% of cases (9.1%) to over 60% of cases (61.3%; Figure 2), representing a 6.7-fold variation from the “best” to “worst.”

## 4. Discussion

This is the first large-scale longitudinal analysis to investigate how variability in testing frequency, as measured by the SD of the HbA1c test interval, relates to the change in HbA1c over time. It is based on data from a total of 83,872 patients with diabetes. We found that, even after adjustment for the number of tests per year, age, sex, and hospital site, the lower variability of the HbA1c test interval (most consistent testing pattern) is associated with a better diabetes control (as defined by *Δ*HbA1c over the 5-year period), compared to those with higher variability in testing frequency (most sporadic testing). This was particularly true for those with a baseline HbA1c of 7.0–9.0% (54–75 mmol/mol), a group where improved blood glucose control is linked to better long-term health outcomes [22–26] but for whom improvement in diabetes control is often a significant challenge. The association was particularly noticeable in those aged under 65 years. We also noted that there was a 6.7-fold variation in proportion of people with the highest variability in testing interval between 216 general practices.

It is important to identify factors that are linked to the HbA1c trajectory as it is well established that higher HbA1c in people with both T1DM and T2DM relates to less favourable long-term health outcomes [22–26]. While we and others have shown that HbA1c testing intervals outside guidance are associated with poorer diabetes control and reduced probability of achieving the target glycaemic status [17, 18], this is the first time that the pattern of testing has been shown to be associated with the trajectory of HbA1c, independently of the test interval and HbA1c level. Thus, for two individuals with the same number of tests per year, our data suggests that the regularity by which those tests are scheduled also appears to be an important determinant of *Δ*HbA1c over time.

Why some individuals appear to be tested for HbA1c more sporadically than others is likely to depend on similar factors to those suggested for the number of tests themselves, namely, those related to (i) healthcare professionals, (ii) the individuals themselves, and (iii) systemic infrastructure factors [27]. We have previously described, at a general practice level in England, that the way that HbA1c and other lab tests testing is organised (i.e., whether a test happens in a given 12-month period) as a manifest in adherence to the nationally recommended care processes [1, 2] is independently related to the proportion of people at any general practice at target glycaemic control. Furthermore, patients of working age may find attending for regular intervals more difficult, as reflected in our observation that a larger proportion of those aged <65 years was in the top-three deciles (i.e., had the most sporadic testing pattern). In terms of systemic factors, the disconnection between primary care (general practices) and secondary (acute hospital) testing may also affect the periodicity in HbA1c testing if one group is unaware of results generated by the other [27].

The reasons underpinning the link between variability in testing interval and HbA1c trajectory are unclear and likely relate to healthcare professionals being more aligned to medication titration if HbA1c is regularly checked and to the way that regularity of testing creates a framework that encourages patient empowerment. This was described by Beard et al. in 2010 [28], who found that only 26.5% of the people with diabetes surveyed had a good understanding of HbA1c and how the level relates to health outcomes. Perception was related to demographics including a number of components of self-care and self-efficacy, as well as HbA1c. The level of understanding was predictive of the HbA1c level. This is relevant because keeping to a regular testing schedule does enhance a sense of self-efficacy [29]. Clearly, as we adjusted for the number of tests, the importance of consistency of monitoring is unlikely to be due to differences in healthcare contacts.

The finding of particularly strong associations in those with a baseline HbA1c in the 7.0–9.0% (54–75 mmol/mol) range is of particular interest. Those with lower values might be argued to require more minimal intervention, whilst those with HbA1c values > 9.0% (>75 mmol/mol) are more likely to be under specialist care due to complex challenges in maintaining diabetes control. However, those in between tend to be managed within general practice and comprise a large cohort of people in whom attempting to improve diabetes control is often a challenge [30]. Therefore, in this group, it is essential to support general practitioners to implement strategies, including ensuring regular monitoring, to help these patients to achieve lower blood glucose levels [30].

Our findings on the differences between general practices showing a 6.7-fold difference in proportion of patients within the top three SD deciles suggest that there is considerable variability in the way in which HbA1c testing is scheduled between practices. Cadogan et al. [16] also demonstrated significant variation between individual general practitioners in requesting of immunoglobulins and showed that this was associated with practitioner experience and sex. However, within a practice comprising several doctors, as measured in our data, this is less likely to be a factor. Our findings also mirror what we have previously shown in terms of practice-level variation in conformity to guidance on testing frequency both in diabetes [6] and other long-term monitoring scenarios [14]. Lyon et al. showed similar variation in HbA1c testing intervals at a regional level [8]. These studies highlight the need for consistency in monitoring people with long-term conditions. While there have been individual attempts to improve HbA1c monitoring [31, 32], including clear recommendations in international guidance [3–5], there appears still some way to go to address this issue. Indeed, using longitudinal laboratory data, we showed that publication of guidance over 8 years made no difference to the proportion of HbA1c tests requested outside guidance [6]. We have no realistic expectation that it will be any different regarding the requesting pattern.

The results of this study are particularly pertinent as we come to appreciate the consequences of the COVID-19 pandemic on the way that routine care for people with diabetes is delivered. In this regard, we recently reported a fall in volume of HbA1c tests following the first UK lockdown in March 2020 [33]. Specifically, by September 2020, there were as many as 1.41 million missed/delayed diabetes monitoring tests, including an estimated 0.51 million in people with suboptimal blood glucose control. As healthcare systems across the world endeavor to catch up in relation to the routine case reviews of people with long-term conditions including diabetes, we have provided evidence for the importance of putting a structured regular HbA1c testing regime in place.

### 4.1. Strengths and Limitations

A significant strength of the paper is the large datasets across two UK regions that we had access to in order to undertake the analysis. However, laboratory data does not have detailed information concerning the duration or type of diabetes, treatment prescribed, comorbidities, or access to expert patient education. Furthermore, data such as ethnicity is inconsistently recorded and therefore unreliable as a covariate.

Our aim with this study was not to elucidate the reason for the different patterns of testing, or its link with HbA1c trajectory, and it is possible that our findings reflect factors or patient/healthcare professional behaviours unrelated to HbA1c testing per se, some of which may have a more direct impact on diabetes control. While we adjusted for the number of HbA1c tests (and hence the number of healthcare professional contacts, to some degree), it is possible that regularity of contacts is indicative of a better within-practice system for long-term monitoring. Youens et al. have recently shown that the regularity of contact with general practitioners, defined as variance in the number of days between contacts, was linked to the diabetes outcome with the most regular decile having fewer hospitalisations and lower per-patient costs than the least regular decile [34]. These data suggest that the pattern as well as frequency of contacts with healthcare professionals may influence patient outcomes.

### 4.2. Summary

Our findings indicated that HbA1c testing consistency, not just numbers of tests/year, is important in maintaining diabetes control, especially in younger (age < 65 years) patients with HbA1c levels in the key 7.0–9.0% (54–75 mmol/mol) range. Furthermore, the consistency of testing also varied between general practices. This has implications for the management of people who attend sporadically for testing and suggests the need for developing systems to improve the regularity of HbA1c testing, particularly in light of the disruption of diabetes services due to the COVID-19 pandemic.

## Figures and Tables

**(a):** 

	PAT		UHNM	
Number of patients	56,809		27,063	
Male (%)	51.5		54.9	
Age (years)^∗^	61	(51, 70)	66	(58, 73)
Baseline HbA1c: (%)^∗^	6.6	(6.0, 7.7)	7.1	(6.5, 8.0)
: (mmol/mol)^∗^	49	(42, 61)	54	(48, 64)
Time between baseline and last test (years)^∗^	4.95	(4.84, 5.06)	4.95	(4.85, 5.06)
Change in HbA1c between baseline and last test: (%)^∗^	−0.1	(−0.5, +0.4)	−0.2	(−0.7, +0.4)
: (mmol/mol)^∗^	−1	(−5, +4)	−2	(−7, +4)
Number of tests^∗^	9	(7, 11)	9	(7, 12)

**(b):** 

Baseline HbA1c; % (mmol/mol)	Number of patients (%)			
	PAT		UHNM	
<5.0 (<30)	218	0.4%	24	0.1%
5.0–5.9 (30–41)	12,679	22.3%	1,281	4.7%
6.0–6.5 (42–47)	12,578	22.1%	4,900	18.1%
6.5–7.0 (48–53)	10,611	18.7%	6,699	24.8%
7.0–7.5 (54–58)	5,589	9.8%	4,370	16.1%
7.5–9.0 (59–75)	8,372	14.7%	6,315	23.3%
9.1–10.0 (76–86)	2,581	4.5%	1,575	5.8%
>10.0 (>86)	4,181	7.4%	1,899	7.0%
*Total*	**56,809**	**100.0%**	**27,063**	**100.0%**

^∗^Results expressed as median (interquartile range). PAT: Pennine Acute Hospitals NHS Trust; UHNM: University Hospitals of North Midlands.

**Table 2:** Association between variability in HbA1c testing intervals and change in HbA1c, by site, sex, and age. Testing interval defined as standard deviation in testing interval for those with at least 6 tests between baseline and 5 years, corrected for the number of tests and stratified by starting HbA1c; linear regression.

Starting HbA1c (mmol/mol (%))	PAT		UHNM		Female		Male		<65 years		≥65 years	
	*p*	Std *β*^∗^	*p*	Std *β*^∗^	*p*	Std *β*^∗^	*p*	Std *β*^∗^	*p*	Std *β*^∗^	*p*	Std *β*^∗^
5.0–5.9 (30–41)	<0.0001	0.10	<0.0001	0.12	<0.0001	0.10	<0.0001	0.09	0.0107	0.04	<0.0001	0.14
6.0–6.5 (42–47)	<0.0001	0.06	<0.0001	0.06	<0.0001	0.06	0.0002	0.05	0.0008	0.04	0.0003	0.05
6.5–7.0 (48–53)	<0.0001	0.06	<0.0001	0.05	<0.0001	0.05	0.0261	0.03	0.1410	0.02	0.0194	0.03
7.0–7.5 (54–58)	<0.0001	0.10	<0.0001	0.09	<0.0001	0.10	<0.0001	0.12	<0.0001	0.08	<0.0001	0.09
7.5–9.0 (59–75)	<0.0001	0.07	<0.0001	0.08	<0.0001	0.06	<0.0001	0.08	0.0033	0.04	<0.0001	0.06
9.1–10.0 (76–86)	<0.0001	0.11	0.0046	0.07	<0.0001	0.10	0.0001	0.09	0.0879	0.06	0.0683	0.05
>10.0 (>86)	0.3146	0.02	0.0755	0.04	0.9448	0.00	0.0178	0.04	0.5444	−0.02	0.4360	0.02

^∗^Standard beta.

## Data Availability

Data are available upon reasonable request from the authors.

## Abbreviations

HbA1c: Glycated haemoglobin
DM: Diabetes mellitus
T1DM: Type 1 diabetes mellitus
T2DM: Type 2 diabetes mellitus
UHNM: University Hospitals of North Midlands NHS Trust
PAT: Pennine Acute Hospitals NHS Trust
SD: Standard deviation.

## Supplementary Materials

Supplementary Materials Supplemental Figure S1: approach used to identify the patient cohort.
