# Supplementary material for: Variability in Test Interval Is Linked to Glycated Haemoglobin (HbA1c) Trajectory over Time
Source: J Diabetes Res. 2022 May 16;2022:7093707. doi: 10.1155/2022/7093707 (PMC9126657; doi:10.1155/2022/7093707)
Supplement: Supplementary Materials — Supplemental Figure S1: approach used to identify the patient cohort. [file 7093707.f1.docx]

**Supplemental Figure 1**

Initial cohort

(3,319,761 tests in 903,667 patients)

Cases with fewer than 6 tests over 5 years

(144,299 tests in 42,637 patients)

Duplicates removed

(1,226,763 tests in 214,656 patients)

Cases with no matching

HbA1c test at 5 years±3months

(1,146.546 tests in 562,502 patients)

Final patient cohort

with at least 6 tests

(802,153 tests in 83,872 patients)

Unique patients with

with baseline and final test

5 years±3months apart

(946,452 tests in 126,509 patients)

Cases with

with test at baseline and

5 years±3months

(2,173,215 tests in 341,165 patients)
